# Editorial: Oxidative Stress in Myocardial and Neural Remodeling

**DOI:** 10.3389/fphys.2021.606484

**Published:** 2021-02-05

**Authors:** Thao P. Nguyen, Sally A. Frautschy, Mansoureh Eghbali

**Affiliations:** ^1^The Cardiovascular Research Laboratory, Division of Cardiology, Department of Medicine, David Geffen School of Medicine at UCLA, Los Angeles, CA, United States; ^2^Department of Neurology, David Geffen School of Medicine at UCLA, Los Angeles, CA, United States; ^3^Department of Anesthesiology and Perioperative Medicine, David Geffen School of Medicine at UCLA, Los Angeles, CA, United States

**Keywords:** oxidative stress, reactive oxygen species, inflammation, structural remodeling, electrical remodeling, cardiac fibrosis, neurodegeneration, Alzheimer's disease

Endogenous oxidative stress arises as an attempted reparative response to injuries, including hypoxia, myocardial infarction, stroke, and radiation-induced phototoxicity. More clinically relevant than ever, oxidative stress has recently been implicated as the key player in the induction of severe acute inflammatory critical illness by the coronavirus SARS-CoV-2 (Delgado-Roche and Mesta, 2020; Wang et al., 2020), the pathogen responsible for the ongoing devastating Covid-19 global pandemic. Oxidative stress occurs when cellular generation of free radicals (reactive oxygen or nitrogen species) overwhelms cellular defense mechanisms. Although oxidative stress serves to rapidly galvanize the immune system into action, direct consequences range from lipid peroxidation, DNA damage, protein misfolding, and mitochondrial dysfunction to inflammation, structural/functional remodeling, and ultimately necrosis. The Research Topic “*Oxidative Stress in Myocardial and Neural Remodeling*” evaluates the damage of oxidative stress specifically to the cardiovascular and nervous systems, defines underlying mechanisms, and proposes novel therapeutic targets.

Starting with the heart, two reviews shed light on the cardiac-chamber specificity in oxidative stress response. Schlüter et al. review evidence that the mitochondrial defense against reactive oxygen species is lower in the right compared with left ventricle. This divergent ventricular response to oxidative stress likely accounts at least in part for the poorer ability of the right ventricle to manage the oxidative stress of pulmonary hypertension (Schlüter et al.). Indeed, in pulmonary hypertension, right ventricular function is the major determinant of survival. Mikhael et al. review evidence that in chronic pulmonary hypertension, oxidative stress induces structural remodeling of not only the pulmonary vasculature but also the right ventricle, eventually causing right heart failure. They discuss why the preclinical success of antioxidant therapies fails to translate clinically (Mikhael et al.).

Delving deeper into the mechanisms of structural remodeling by oxidative stress, Li et al. delineate a molecular pathway linking oxidative stress, diet, and inflammation with myocardial fibrosis and identify a novel potential target for antifibrotic therapy. Using mouse models of doxorubicin-induced cardiomyopathy, they demonstrate how trimethylamine N-oxide (TMAO), a gut microbiota-dependent metabolite of specific dietary nutrients, can aggravate myocardial fibrosis through activation of the Nucleotide-binding oligomerization domain (NOD)-like receptor protein 3 (NLRP3) inflammasome (Li et al.).

Zhao et al., on the other hand, investigated the mechanistic role of oxidative stress in electrical remodeling, particularly during synergy with existing structural and electrical remodeling by noncardiomyocytes, including myofibroblasts (Zhao et al.). Using a novel *in-vitro* two-dimensional model of interface between cardiomyocytes and noncardiomyocytes that they developed, the authors demonstrated a functional proof-of-concept for the contribution of oxidative stress to the dual protective and proarrhythmic roles of myofibroblasts and other noncardiomyocytes. Not only connexin43-rich noncardiomyocytes can enable passive, decremental, anisotropic impulse propagation, but they can also, independently or synergistically with oxidative stress, induce new arrhythmia triggers, dynamic functional conduction block, non-reentrant and reentrant arrhythmias.

The next three articles examine how oxidative stress causes adverse structural remodeling in both the cardiac and central nervous systems concomitantly. Carbone et al. emphasize the critical role of neutrophils in cardiac and neural remodeling mediated by the oxidative stress of ischemia-reperfusion injury as neutrophils are activated early for massive production of reactive oxygen species. The authors discuss how anatomical and functional differences between these two organs shape their responses to oxidative stress and suggest that attention to these differences may improve translational success of preclinical trials (Carbone et al.). Supporting Carbone et al.'s conclusion, Vaillancourt et al. demonstrate that the activities of reactive oxygen and nitrogen species may extend beyond local tissues. They discover how oxidative stress in pulmonary hypertensive rats induces neuroinflammation remotely in the thoracic spinal cord (Vaillancourt et al.). Denver et al., on the other hand, explore the mechanistic synergism between coexisting oxidative stress in heart and brain and the resultant clinical manifestations. Based on the insight that the central nervous system vasculature is particularly vulnerable to oxidative stress (Bennett et al., 2009; He et al., 2020), the authors develop a novel, ingenious rat model of human mixed vascular dementia by compounding the oxidative stress induced by the transgene of Alzheimer's disease with that induced by the phenotype of chronic hypertension. They discover that hypertension exacerbates the neuroinflammation of Alzheimer's dementia, leading to new fibrotic remodeling and ensuing pathology for the brain vasculature (Denver et al.).

Exploring yet another vasculature system, Ishiyama et al. discover that in Ménière's disease, endogenous oxidative stress primarily targets the vascular endothelial cells of the blood labyrinthine barrier for structural remodeling. The inflammation triggered by oxidative stress causes pericytes to degenerate and migrate, thereby compromising the structural integrity of the blood labyrinthine barrier and its critical function in ionic and fluid homeostasis (Ishiyama et al.).

Moving from pathophysiology to therapeutics, Deres et al. conduct a test of the efficacy and safety of a novel anti-inflammatory drug, bradykinin B1 receptor antagonist FGY-1153 in spontaneously hypertensive rats. The authors conclude that this novel agent provides moderate protection against the development of hypertensive cardiomyopathy and has no cardiovascular toxicities (Deres et al.). However, additional randomized preclinical and clinical trials are necessary to confirm these promising findings regarding the efficacy and safety profile of FGY-1153.

Taken together, the collection of original research and review articles presented in this Research Topic provides an overview and update of insights into the critical role of oxidative stress on myocardial and neural remodeling. We hope that this collective knowledge would inspire and drive further research for innovative and effective anti-oxidative therapeutic strategies to reduce the burden of morbidity and disability due to oxidative stress.

## Author Contributions

TN conceptualized this Research Topic, recruited co-editors, and wrote the original draft. All authors contributed to manuscript revision, approved the submitted version, and agreed to be accountable for all aspects of the work.

## Conflict of Interest

The authors declare that the research was conducted in the absence of any commercial or financial relationships that could be construed as a potential conflict of interest.
